# Impact of dialysis modality choice on the survival of end-stage renal disease patients with congestive heart failure in southern China: A retrospective cohort study

**DOI:** 10.3389/fmed.2022.898650

**Published:** 2022-10-17

**Authors:** Zhiren He, Hui Liang, Jing Huang, Defei Zhang, Hongyan Ma, Junjie Lin, Youqing Cai, Tonghuan Liu, Hucai Li, Weizhong Qiu, Lingzheng Wang, Fengling Yuan, Haijing Hou, Daixin Zhao, Xusheng Liu, Lixin Wang

**Affiliations:** ^1^Department of Nephrology, The Second Affiliated Hospital of Guangzhou University of Chinese Medicine, Guangzhou, Guangdong, China; ^2^The Second Clinical College of Guangzhou University of Chinese Medicine, Guangzhou, Guangdong, China

**Keywords:** all-cause mortality, hemodialysis, peritoneal dialysis (PD), end-stage renal disease (ESRD), congestive heart failure (CHF)

## Abstract

**Background and object:**

Heart failure is one of the common complications in patients with end-stage renal disease (ESRD) and a major cause of death in these patients. The choice of dialysis modality for ESRD patients with congestive heart failure (CHF) is still inconclusive. The purpose of this study was to compare the prognosis of hemodialysis (HD) and peritoneal dialysis (PD) among ESRD patients with CHF and provide a basis for clinical decision-making.

**Materials and methods:**

This was a retrospective study conducted at Guangdong Provincial Hospital of Traditional Chinese Medicine that included patients with CHF requiring long-term renal replacement therapy between January 1, 2012 and December 31, 2017. The end of follow-up was December 31, 2020. All patients were divided into HD and PD groups and sub grouped by age, and we used univariate and multifactorial Cox regression analyses to calculate the relative hazard ratios (HR) of the different dialysis types and adjusted for differences in baseline data using propensity score matching (PSM).

**Result:**

A total of 121 patients with PD and 156 patients with HD were included in this study. Among younger ESRD patients (≤65 years of age) with CHF, the prognosis of HD was worse than that of PD [HR = 1.84, 95% confidence interval (CI) = 1.01–3.34], and this disadvantage remained significant in the fully adjusted model [sex, age at dialysis initiation, Charlson comorbidities index, body mass index, prealbumin, hemoglobin, and left ventricular ejection fraction (LVEF)] and after PSM. In the older group (>65 years of age), the prognosis of HD was better than that of PD (HR = 0.46, 95% CI = 0.25–0.85), and the protective effect remained in the fully adjusted model and after PSM. The aforementioned survival differences across the cohort were maintained in patients with preserved LVEF (>55%), but could not be reproduced in patients with reduced LVEF (≤55%).

**Conclusion:**

In southern China, PD is a better choice for younger patients with ESRD, CHF and preserved LVEF, and HD is the better option for older patients.

## Introduction

Previous studies have shown that 14.7–33% of patients have congestive heart failure (CHF) when they start dialysis (1–5) and are repeatedly hospitalized for CHF during subsequent treatments (6). Heart failure is widespread in maintenance dialysis patients, and dialysis patients with CHF have a higher risk of death than those without CHF (7–9). The most common reason for heart failure in these patients is volume overload with or without heart diseases (10).

Both hemodialysis (HD) and peritoneal dialysis (PD) are believed to reduce volume overload and improve heart failure through ultrafiltration. After PD was developed, it was often used to treat heart failure in non-ESRD patients. A number of reports have shown that PD has a good effect on refractory heart failure (11, 12). However, it is unclear which dialysis method has the better prognosis among ESRD dialysis patients with CHF. Only a small number of studies have investigated the prognostic differences in patients with CHF after receiving PD or HD. These studies have found that at the beginning of dialysis, patients with CHF may have different prognoses for different dialysis treatments. All of them are retrospective cohort studies (1–3, 5, 13). PD is generally considered to have a poor prognosis (1–3, 6). However, some recent studies have suggested that HD and PD may be equally effective (5, 13).

The many factors that affect the choice of dialysis methods make it almost impossible to conduct randomized controlled trials to evaluate the pros and cons of the two dialysis methods (14). In different regions and at different times, ESRD patients with heart failure choose different dialysis methods, and their prognoses are different. China is a country with a large population, and a large number of patients receiving renal replacement therapy also have CHF. However, there is currently a lack of clinical studies evaluating the impact of HD and PD on the prognosis of these patients. Thus, clinicians and patients lack clinical evidence to support the choice of dialysis methods.

China has been conducting nationwide registration of HD and PD patient information in recent years, but the accuracy of the registration information needs to be improved. We have established a relatively complete follow-up system to monitor the prognosis of renal replacement therapy patients.

Previous studies at our center have shown that the prognoses of PD and HD are different for end-stage renal patients at different ages (15). We hypothesize that the prognosis of ESRD patients with CHF receiving different dialysis methods may be different. Therefore, a subgroup analysis of the original data was carried out to compare the all-cause mortality of dialysis patients with CHF and to evaluate the impact of the two dialysis methods on the prognosis of such patients. Our findings will provide a reference for clinicians and patients to make decisions.

## Materials and methods

### Populations

A retrospective cohort study was conducted from January 1, 2012 to December 31, 2017, in the Nephrology Department of Guangdong Provincial Hospital of Chinese Medicine. The inclusion criteria were ESRD patients with CHF aged older than 18 years at dialysis start in need of long-term maintenance of renal replacement therapy as judged by clinicians. The patients’ CHF diagnosis was determined by the diagnosis code at the time of initiation of dialysis. The exclusion criteria were a lack of baseline data, combined renal replacement therapy (both HD and PD), and malignant disease. The outcome events were assessed from previous follow-up records.

### Covariates

Baseline demographics, comorbid conditions, laboratory test results, echocardiographic measurement results, and hospitalization events were obtained by reviewing the electronic medical records. The demographic data included the date of birth, sex, start of dialysis, primary onset of kidney disease, height, and weight at dialysis start. The comorbidities and the New York Heart Association (NYHA) heart functional classification were identified at baseline medical records according to the International Classification of Diseases, 9th and 10th Revision (ICD-9 and ICD-10) codes, and the Charlson comorbidity index (CCI) score was calculated based on Quan et al.’s method (16). Cardiovascular diseases included asymptomatic myocardial ischemia (occult coronary heart disease), angina pectoris, myocardial infarction, and ischemic heart failure (ischemic heart disease). Heart failure with reduced left ventricular ejection fraction (HFrEF) was defined as left ventricular ejection fraction (LVEF) less than or equal to 55%. Heart failure with preserved LVEF (HFpEF) was defined as LVEF over 55%.

### Outcomes

The main outcome was all-cause mortality of the patient. The censor event included the patient switching to another dialysis mode, undergoing a kidney transplant, transferring to another dialysis center to continue treatment, or reaching the end of follow-up (December 31, 2020). We checked and registered the patient’s cause of death through the death registration system of the Chinese Center for Disease Control and Prevention.

### Statistical methods

Furthermore, medical records were collected to deduce the statistical significance of the results. According to Hsieh and Lavori’s method (17), α was set to 0.05 and *p* to 0.95, the estimated hazard ratio was 0.5, the proportion of the event was 0.34, the proportion of withdrawals was 0.24, the standard deviation of interest covariate was 0.5, the correlation of covariates was 0, the estimated cohort size was 240, and the number of events was 62.

Baseline characteristics and laboratory tests of HD patients were compared to those of PD patients. Normally distributed continuous variables are presented as the mean ± standard deviation (SD), and *t*-tests were used for comparison. Skewed data are presented as the median and rank, and the Mann–Whitney *U* test was used for comparisons between groups. Missing data were filled in using a multiple imputation by chained equations algorithm by using R’s MICE package. The filling method was “mean”. Categorical variables are presented as percentages and were analyzed by the chi-square test.

The Kaplan-Meier survival curve was used to compare the overall survival between the initial dialysis modalities, and the significance of the difference was tested by the log-rank method. Previous studies (18, 19) have shown that PD has an advantage in the first 2 years of dialysis. Therefore, we used 2 years as the boundary to observe short-term and long-term prognosis.

A Cox regression model was used to perform multivariate analysis. The covariates of the multivariate regression were also selected based on univariate regression result, clinical experience and previous studies and included sex, age at dialysis initiation, CCI score, LVEF, prealbumin (PA), and hemoglobin (HB). Using too many variables could lead to overfitting bias in the Cox regression model due to insufficient end-point events. We therefore used a stepwise process by using R’s My.stepwise package for variable selection for each model. The final variables selected for each model will be displayed in the results.

We also used propensity score matching (PSM) to reduce the effect of selection bias. We used the MatchIt package in R for PSM at a ratio of 1:1. The matching method was nearest neighbor matching. The characteristics used in PSM were sex, age at dialysis initiation and CCI score for the all groups. Subgroup analyses were performed with respect to age. Previous studies (15) suggested that patients undergoing HD and PD have different prognoses in different age subgroups. Therefore, we used a Cox regression model to confirm the interactive effect of age and dialysis type. Further analysis of the interaction effects suggested that 65 years old may be the cut-off point for the difference in the prognosis of different dialysis methods. We found that in the subgroups of patients ≤ 65 years old and >65 years old, the type of dialysis had a significant effect on the prognosis. Patients were grouped by age. Then, single-factor and multivariate Cox regression analyses were performed to calculate the relative hazard ratio (HR) of the dialysis types, followed by PSM and multivariate Cox regression analyses to confirm the relative HR of the dialysis types.

Subgroup analyses were also performed with respect to LVEF. Previous studies (20–22) suggested that patients with HFrEF had a poorer prognosis. We used Kaplan-Meier survival curve and Cox regression model to compare the survival difference between patients ≤ 65 years old and >65 years old.

We used the Fine and Gray competing risk regression model (23) by using R’s cmprsk package to calculate the hazard ratios of cardiovascular and cerebrovascular death and death from infection in different age subgroups of patients with different dialysis methods.

All statistical tests were evaluated using a two-tailed 95% confidence interval (CI), and *p* < 0.05 was considered indicative of statistical significance. All statistical analyses were performed using the R language (version 3.6.0) or Python lifelines package (version 0.26.4).

## Results

### The baseline characteristics of the patients

**FIGURE 1:**
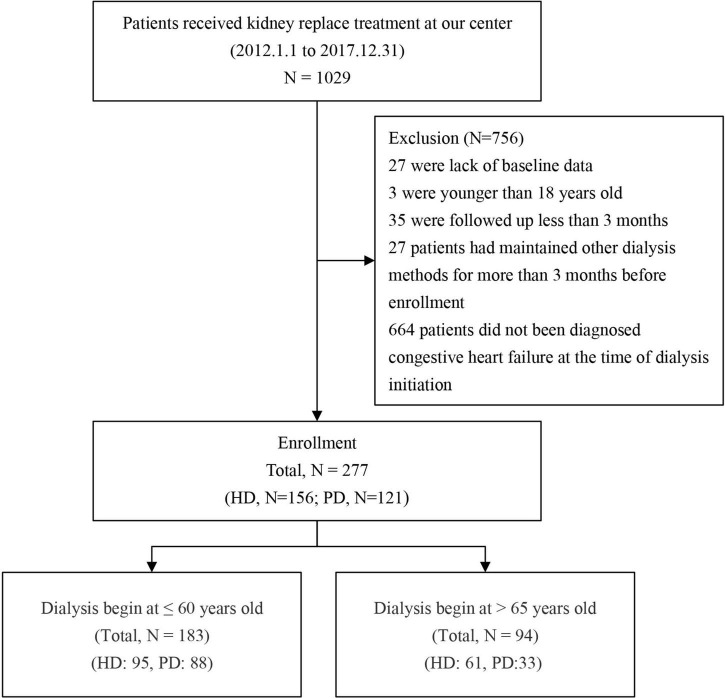
Study flow diagraph.

This study included all patients who began dialysis treatment in Guangdong Hospital of Traditional Chinese Medicine from January 1, 2012 to December 31, 2017. According to the principle of inclusion and exclusion, 277 patients were included and divided into two groups according to the dialysis method: there were 121 patients in the PD group and 156 in the HD group (Figure 1).

Demographic and clinical features are shown in Table 1. The average age at dialysis initiation in the HD group was older than that in the PD group (54.18 ± 16.06 vs. 61.40 ± 14.44, *p* < 0.05), and the average follow-up time of the HD group was also longer than that of the PD group (30.88 ± 18.29 vs. 36.01 ± 20.94, *p* = 0.03). There were more female patients in the HD group (37.19 vs. 53.21% *p* = 0.01). The rates of diabetes and cardiovascular disease were similar between the two groups. This finding was observed in all age groups.

**TABLE 1:** Baseline characteristics.

	All patients			Patients younger than 65 years old			Patients older than 65 years old		
			
	PD group (*n* = 121)	HD group (*n* = 156)	*P*	PD group (*n* = 88)	HD group (*n* = 95)	*P*	PD group (*n* = 33)	HD group (*n* = 61)	*P*
**Demographic data**									
Female (*n*)	45 (37.19%)	83 (53.21%)	0.01	30 (34.09%)	45 (47.37%)	0.09	15 (45.45%)	38 (62.30%)	0.18
Age of dialysis initiation (years)	54.18 ± 16.06	61.40 ± 14.44	0.00	47.00 ± 12.45	52.66 ± 11.11	0.00	73.33 ± 5.13	75.02 ± 5.99	0.18
Body mass index (kg/m^2^)	22.67 ± 4.53	24.09 ± 4.06	0.01	22.27 ± 3.19	24.20 ± 4.11	0.00	23.75 ± 6.90	23.93 ± 4.01	0.87
Duration of follow up (months)	41.77 ± 25.07	48.16 ± 27.19	0.05	46.23 ± 26.09	47.61 ± 26.81	0.73	29.88 ± 17.50	49.01 ± 27.96	0.00
**Kidney primary disease**									
Diabetic nephropathy (*n*)	60 (49.59%)	57 (36.54%)	0.04	42 (47.73%)	36 (37.89%)	0.23	18 (54.55%)	21 (34.43%)	0.09
Glomerulonephritis (*n*)	35 (28.93%)	29 (18.59%)	0.06	28 (31.82%)	24 (25.26%)	0.41	7 (21.21%)	5 (8.20%)	0.14
Polycystic kidney (*n*)	1 (0.83%)	3 (1.92%)	0.80	0 (0.00%)	2 (2.11%)	0.51	1 (3.03%)	1 (1.64%)	0.76
Obstructive nephropathy (*n*)	5 (4.13%)	11 (7.05%)	0.44	3 (3.41%)	6 (6.32%)	0.57	2 (6.06%)	5 (8.20%)	0.97
Other or unknown (*n*)	20 (16.53%)	56 (35.90%)	0.00	15 (17.05%)	27 (28.42%)	0.10	5 (15.15%)	29 (47.54%)	0.00
**Comorbidities**									
CCI	6.42 ± 2.33	7.24 ± 2.07	0.00	5.58 ± 2.03	6.33 ± 1.97	0.01	8.67 ± 1.41	8.67 ± 1.26	0.98
Diabetes (*n*)	65 (53.72%)	85 (54.49%)	1.00	43 (48.86%)	50 (52.63%)	0.72	22 (66.67%)	35 (57.38%)	0.51
Cardiovascular disease (*n*)	30 (24.79%)	46 (29.49%)	0.46	15 (17.05%)	21 (22.11%)	0.50	15 (45.45%)	25 (40.98%)	0.84
Cerebrovascular disease (*n*)	12 (9.92%)	33 (21.15%)	0.02	5 (5.68%)	11 (11.58%)	0.25	7 (21.21%)	22 (36.07%)	0.21
Chronic pulmonary disease (*n*)	10 (8.26%)	12 (7.69%)	0.96	6 (6.82%)	7 (7.37%)	0.89	4 (12.12%)	5 (8.20%)	0.80
**Cardiac function evaluation**									
LVEF (%)	60.12 ± 12.17	60.49 ± 10.72	0.79	58.78 ± 12.42	59.87 ± 10.19	0.52	63.70 ± 10.84	61.46 ± 11.52	0.36
NYHA III (*n*)	62 (51.24%)	64 (41.03%)	0.12	42 (47.73%)	36 (37.89%)	0.23	20 (60.61%)	28 (45.90%)	0.25
NYHA IV (*n*)	21 (17.36%)	28 (17.95%)	0.98	16 (18.18%)	19 (20.00%)	0.90	5 (15.15%)	9 (14.75%)	0.80
**Laboratory tests**									
Serum urea (mmol/l)	23.35 ± 11.86	21.42 ± 11.46	0.17	22.98 ± 11.32	22.50 ± 12.33	0.78	24.33 ± 13.32	19.74 ± 9.82	0.09
Serum creatinine (μmol/l)	777.72 ± 286.91	697.97 ± 325.94	0.03	805.80 ± 274.55	747.85 ± 337.46	0.20	702.84 ± 309.52	620.30 ± 293.19	0.20
Triglyceride (mmol/L)	1.31 ± 0.71	1.40 ± 1.06	0.47	1.36 ± 0.65	1.48 ± 1.27	0.43	1.20 ± 0.84	1.27 ± 0.59	0.66
Cholesterol (mmol/L)	4.60 ± 1.58	4.40 ± 1.13	0.22	4.69 ± 1.59	4.37 ± 1.18	0.12	4.38 ± 1.54	4.45 ± 1.05	0.82
LDLC (mmol/L)	2.88 ± 1.36	2.66 ± 0.96	0.13	2.95 ± 1.37	2.63 ± 1.01	0.08	2.71 ± 1.32	2.71 ± 0.88	0.99
HDLC (mmol/L)	1.07 ± 0.30	1.08 ± 0.33	0.96	1.07 ± 0.30	1.07 ± 0.33	1.00	1.07 ± 0.29	1.08 ± 0.32	0.94
Plasma albumin (g/L)	33.23 ± 4.67	34.74 ± 5.30	0.01	32.87 ± 4.61	34.70 ± 5.68	0.02	34.20 ± 4.77	34.80 ± 4.71	0.56
Prealbumin (g/L)	286.50 ± 90.70	262.10 ± 88.78	0.03	302.48 ± 91.56	276.73 ± 98.64	0.07	243.89 ± 74.02	239.31 ± 65.21	0.76
Hemoglobin (g/L)	79.68 ± 18.89	85.88 ± 21.59	0.01	81.42 ± 19.59	85.95 ± 20.34	0.13	75.03 ± 16.24	85.77 ± 23.58	0.02
Phosphorus (mmol/L)	1.80 ± 0.64	1.76 ± 0.61	0.52	1.85 ± 0.64	1.89 ± 0.65	0.70	1.68 ± 0.62	1.55 ± 0.50	0.26
Calcium (mmol/L)	2.05 ± 0.30	2.06 ± 0.28	0.65	2.02 ± 0.28	2.04 ± 0.27	0.62	2.13 ± 0.35	2.10 ± 0.28	0.71
**Cause of death**									
Cardiovascular cause (*n*)	11 (9.09%)	21 (13.46%)	0.35	5 (5.68%)	11 (11.58%)	0.25	6 (18.18%)	10 (16.39%)	0.95
Infectious disease (*n*)	9 (7.44%)	11 (7.05%)	0.91	3 (3.41%)	2 (2.11%)	0.93	6 (18.18%)	9 (14.75%)	0.89
Intracerebral hemorrhage (*n*)	1 (0.83%)	9 (5.77%)	0.06	1 (1.14%)	6 (6.32%)	0.15	0 (0.00%)	3 (4.92%)	0.50
Other or unknown (*n*)	13 (10.74%)	20 (12.82%)	0.73	7 (7.95%)	14 (14.74%)	0.23	6 (18.18%)	6 (9.84%)	0.40

PD, peritoneal dialysis; HD, hemodialysis; LDLC, low-density lipoprotein; HDLC, high-density lipoprotein; CCI, Charlson Comorbidities Index; LVEF, left ventricular ejection fraction; NYHA, New York Heart Association.

In terms of primary renal disease, the proportions of polycystic kidney disease, obstructive nephropathy and glomerulonephritis were similar between the two groups. The proportion of diabetic nephropathy in the PD group was significantly higher than that in the HD group (49.59 vs. 36.54% *p* = 0.04), but among the age subgroups, this difference was not significant.

In terms of complications, patients in the HD group were more likely to have cerebrovascular diseases (9.92 vs. 21.15% *p* = 0.02), and the CCI value was also higher (6.42 ± 2.33 vs. 7.24 ± 2.07 *p* < 0.05). In terms of laboratory examinations, compared with those in the HD group, serum creatinine and prealbumin were higher, and plasma albumin and hemoglobin were lower in the PD group at the beginning of dialysis. Moreover, plasma albumin was lower in PD group patients less than 65 years old. In patients older than 65 years old, hemoglobin in the PD group was still lower.

### Survival difference between the peritoneal dialysis and hemodialysis

**FIGURE 2:**
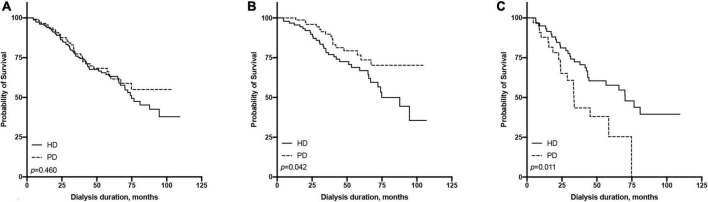
Comparison of survival rate between hemodialysis and peritoneal dialysis by age subgroups [**(A)** all patients, **(B)** ≤65 years old group, **(C)** >65 years old group].

The 1-, 3-, and 5-year survival rates were 96.01, 75.92, and 63.17% in the PD group and 96.53, 77.39, and 61.57% in the HD group, respectively (Table 2). The Kaplan-Meier survival analysis showed that the overall prognosis of the two dialysis methods varied but not significantly (*p* = 0.460, Figure 2A).

**TABLE 2:** Cohort outcomes.

	All-cause mortality	Person-years	One year survival rate	Two years survival rate	Five years survival rate	Mortality rate (dead/1,000 person-years)	Mortality rate ratio (95% CI)
**All-patients**							
HD (*n* = 156)	61	617.47	96.05%	75.92%	63.17%	98.79	1.21 (0.78–1.89)
PD (*n* = 121)	34	415.42	96.53%	77.39%	61.57%	81.84	
**Age ≤ 65 years old**							
HD (*n* = 95)	33	371.72	96.74%	78.24%	66.85%	88.78	1.86 (0.99–3.60)
PD (*n* = 88)	16	334.38	100.00%	89.82%	73.60%	47.85	
**Age > 65 years old**							
HD (*n* = 61)	28	245.75	94.97%	72.41%	57.71%	113.94	0.51 (0.27–0.98)
PD (*n* = 33)	18	81.04	87.88%	43.44%	25.34%	222.11	

PD, peritoneal dialysis; HD, hemodialysis; 95% CI, 95% confidence interval.

**FIGURE 3:**
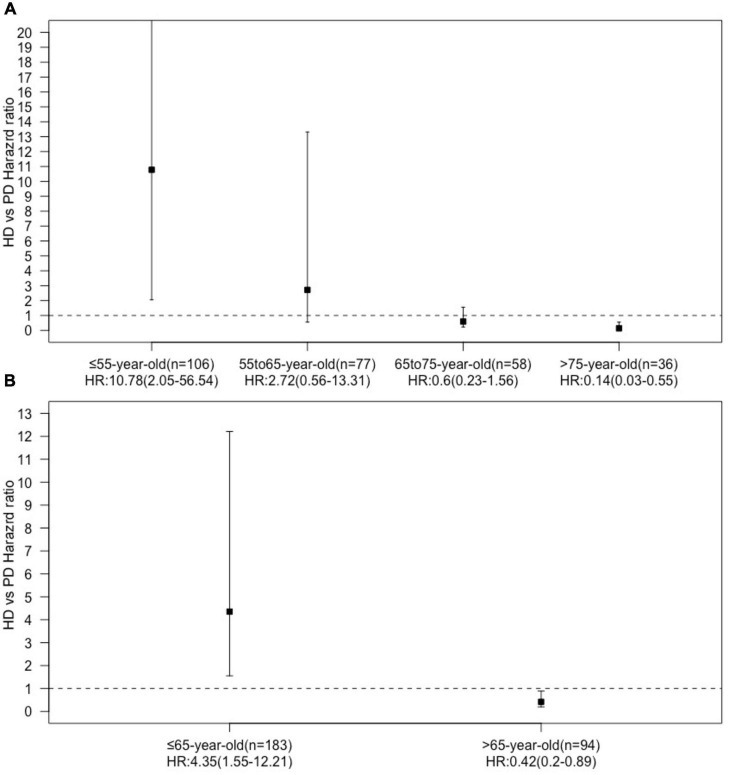
Hazard ration dialysis methods (HD vs. PD) in age subgroups [**(A)** four age subgroups, **(B)** two age subgroups].

We found an interaction between the age at dialysis initiation and dialysis modality. Therefore, we analyzed the prognosis of patients of different ages at dialysis initiation. We found that in the multivariate Cox regression model, the HR of HD vs. PD increased with the age at dialysis initiation (Figure 3). We finally set 65 years old as the cut-off point for the difference in the prognosis of different dialysis modalities.

All patients were divided into two groups based on the age of dialysis initiation (≤65 and >65 years old). In the above age groups, the all-cause mortality rate ratios of HD vs. PD were 1.86 (95% CI: 0.99–3.60) and 0.51 (95% CI: 0.27–0.98), respectively (Table 2). In the ≤65-year-old subgroup, the 1-, 3-, and 5-year survival rates of the PD group were 100.00, 89.82, and 73.60%, while those of the HD group were 96.74, 78.24, and 66.85%, respectively. The log-rank test showed that survival in the PD group was significantly higher than that in the HD group (*p* = 0.042, Figure 2B). In the >65-year-old subgroup, the 1-, 3-, and 5-year survival rates in the PD group were 87.88, 43.44, and 25.34%, and those in the HD group were 94.97, 72.41, and 57.71%, respectively, indicating that HD had a better prognosis in this age group, which was statistically significant (*p* = 0.011, Figure 2C).

### Short- and long-term survival differences between peritoneal dialysis and hemodialysis

**FIGURE 4:**
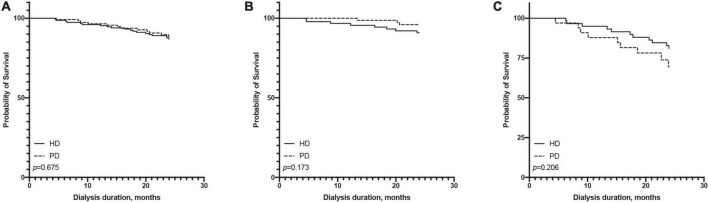
Comparison of first 2 years survival rate between hemodialysis and peritoneal dialysis by age subgroups [**(A)** all patients, **(B)** ≤65 years old group, **(C)** >65 years old group].

**FIGURE 5:**
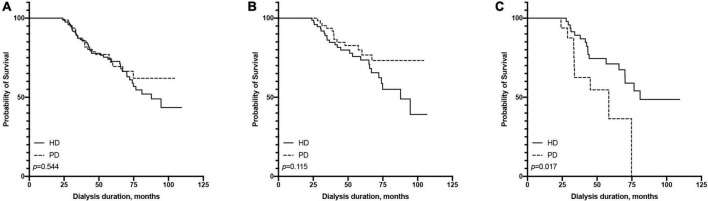
Comparison of pass 2 years survival rate between hemodialysis and peritoneal dialysis by age subgroups [**(A)** all patients, **(B)** ≤65 years old group, **(C)** >65 years old group].

In terms of short-term survival (the first 2 years of follow-up), PD showed a tendency of a survival advantage in the ≤65-year-old subgroup, and HD showed a tendency of a survival advantage in the >65-year-old subgroup; however, neither reached statistical significance (Figure 4). In terms of long-term survival (after the first 2 years of followup), PD showed a survival advantage in the ≤65-year-old subgroup, but it did not reach statistical significance (*p* = 0.115). HD showed a significant survival advantage in the >65-year-old subgroup (*p* = 0.017, Figure 5).

### Survival differences between hemodialysis and peritoneal dialysis in the subgroup of patients with HFrEF (≤55%) and HFpEF (>55%) at start of dialysis

**FIGURE 6:**
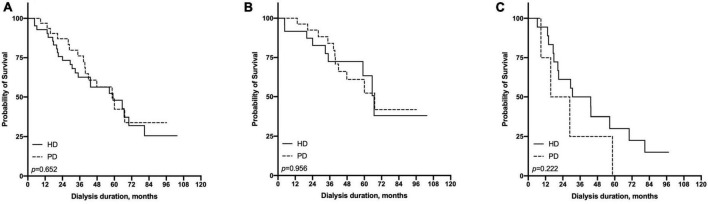
Comparison of survival rate between HD and PD in the subgroup of patients with reduce LVEF (≤55%) [**(A)** all patients, **(B)** ≤65 years old group, **(C)** >65 years old group].

**FIGURE 7:**
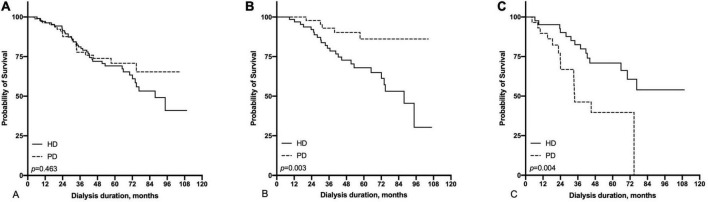
Comparison of survival rate between HD and PD in the subgroup of patients with preserved LVEF (>55%) [**(A)** all patients, **(B)** ≤65 years old group, **(C)** >65 years old group].

In the subgroup with HFrEF, the survival advantage of PD in younger patients and HD in the elderly subgroup disappeared (Figure 6). In the subgroup with HFpEF, PD showed better survival in the ≤65-year-old subgroup (*p* = 0.003), and HD showed a significantly higher survival in the >65-year old subgroup (*p* = 0.004, Figure 7).

### Hazard ratio of factors associated with all-cause mortality

For the entire follow-up period, in the ≤65-year-old subgroup, the univariate Cox regression model suggested that diabetes, the CCI score, cardiovascular disease, triglycerides, and HD were risk factors for all-cause mortality (Table 3). The multivariate Cox regression model suggested that cardiovascular disease, cholesterol, low-density lipoprotein cholesterol (LDLC), and HD were risk factors, and they were still risk factors after using PSM to eliminate the differences in baseline characteristics. The All-cause mortality HR of HD vs. PD increased from 1.84 (95% CI: 1.01–3.34) to 2.48 (95% CI: 1.23–4.99). And the HR remained significant in stepwise model (Table 4). In the >65-year-old subgroup, the univariate and multivariate Cox regression models suggested that HD was a protective factor for all-cause mortality. The HR decreased from 0.46 (95% CI: 0.25–0.85) to 0.28 (95% CI: 0.14–0.57) (Table 4). After using PSM to eliminate the differences in baseline characteristics, HD was still a protective factor for multivariate Cox regression (Table 4).

**TABLE 3:** Risk factors for all-cause mortality assessed by univariate Cox regression model.

	Patients younger than 65 years old PD (*n* = 88), HD (*n* = 95)			Patients older than 65 years old PD (*n* = 33), HD (*n* = 61)		
		
	Entire group HR (95% CI)	HFrEF group (PD:30, HD:25) HR (95% CI)	HFpEF group (PD:58, HD:70) HR (95% CI)	Entire group HR (95% CI)	HFrEF group (PD:4, HD:18) HR (95% CI)	HFpEF group (PD:29, HD:43) HR (95% CI)
Age at dialysis initiation (per 10 year)	1.24 (0.96–1.60)	0.98 (0.71–1.37)	1.52 (1.04–2.21)	1.34 (0.85–2.11)	1.16 (0.64–2.10)	1.33 (0.71–2.51)
Sex (female vs. male)	0.74 (0.42–1.32)	0.63 (0.24–1.65)	0.90 (0.43–1.86)	0.88 (0.49–1.57)	1.23 (0.47–3.19)	0.75 (0.35–1.57)
Diabetes (*n*)	2.49 (1.36–4.54)	1.97 (0.81–4.84)	3.46 (1.47–8.16)	1.12 (0.62–2.03)	0.82 (0.31–2.15)	1.20 (0.56–2.56)
CCI (per 1 point)	1.29 (1.07–1.55)	1.36 (0.99–1.88)	1.31 (1.03–1.67)	0.95 (0.76–1.20)	0.71 (0.41–1.23)	0.95 (0.71–1.25)
Cerebrovascular disease (*n*)	1.00 (0.36–2.79)	1.73 (0.39–7.62)	0.79 (0.19–3.33)	1.09 (0.59–2.00)	0.84 (0.31–2.29)	1.19 (0.54–2.59)
Chronic pulmonary disease (*n*)	0.91 (0.28–2.94)	0.86 (0.11–6.56)	0.96 (0.23–4.04)	1.19 (0.47–3.01)	1.38 (0.44–4.27)	0.45 (0.06–3.33)
Cardiovascular disease (*n*)	3.89 (2.16–6.99)	2.35 (0.97–5.69)	5.20 (2.29–11.80)	1.48 (0.83–2.65)	0.68 (0.25–1.79)	1.64 (0.78–3.45)
BMI (per 1 kg/m^2^)	1.02 (0.95–1.09)	1.02 (0.92–1.13)	1.03 (0.94–1.12)	0.94 (0.87–1.01)	0.99 (0.94–1.04)	0.85 (0.76–0.95)
LVEF (per 10%)	0.73 (0.59–0.90)	0.79 (0.51–1.22)	0.59 (0.33–1.06)	0.71 (0.57–0.89)	0.88 (0.48–1.59)	0.70 (0.38–1.29)
Hemoglobin (per 10 g/L)	1.06 (0.93–1.22)	1.07 (0.86–1.32)	1.07 (0.89–1.28)	1.03 (0.91–1.16)	1.18 (0.96–1.45)	0.98 (0.84–1.15)
Prealbumin (per 50 g/L)	0.88 (0.76–1.03)	0.86 (0.66–1.12)	0.90 (0.74–1.08)	0.93 (0.73–1.17)	1.28 (0.88–1.86)	0.82 (0.59–1.13)
Plasma albumin (per 5 g/L)	0.86 (0.65–1.12)	0.74 (0.49–1.13)	0.91 (0.65–1.29)	0.92 (0.70–1.22)	0.97 (0.58–1.60)	0.96 (0.68–1.35)
Triglyceride (per 1 mmol/L)	1.17 (1.03–1.32)	2.38 (1.41–4.00)	1.16 (0.99–1.35)	1.08 (0.68–1.72)	2.06 (0.90–4.71)	0.94 (0.50–1.76)
Cholesterol (per 1 mmol/L)	1.17 (0.97–1.42)	1.30 (1.03–1.63)	1.08 (0.82–1.42)	1.07 (0.82–1.40)	1.16 (0.69–1.94)	1.13 (0.81–1.57)
LDLC (per 1 mmol/L)	1.17 (0.94–1.45)	1.32 (1.02–1.72)	1.04 (0.75–1.43)	1.18 (0.85–1.63)	1.16 (0.62–2.15)	1.31 (0.89–1.95)
HDLC (per 1 mmol/L)	0.33 (0.12–0.90)	0.45 (0.09–2.18)	0.31 (0.09–1.12)	0.63 (0.22–1.78)	0.96 (0.23–4.09)	0.32 (0.07–1.40)
Phosphorus (per 1 mmol/L)	0.66 (0.42–1.03)	0.63 (0.34–1.18)	0.73 (0.40–1.36)	0.92 (0.55–1.55)	1.29 (0.53–3.13)	0.85 (0.43–1.67)
Calcium (per 1 mmol/L)	0.87 (0.32–2.38)	0.45 (0.12–1.64)	1.80 (0.42–7.78)	1.36 (0.50–3.65)	2.65 (0.57–12.27)	0.98 (0.26–3.70)
Dialysis Methods (HD *vs.* PD)	1.84 (1.01–3.34)	1.03 (0.42–2.48)	3.83 (1.46–10.05)	0.46 (0.25–0.85)	0.50 (0.16–1.56)	0.33 (0.15–0.72)

HR, hazard ratio; 95% CI, 95% confidence interval; PD, peritoneal dialysis; HD, hemodialysis; CCI, Charlson Comorbidities Index; BMI, body mass index; LDLC, low-density lipoprotein; HDLC, high-density lipoprotein; LVEF, left ventricular ejection fraction; HFrEF, heart failure with reduced LVEF (≤55%); HFpEF, Heart failure with preserved LVEF (>55%).

**TABLE 4:** Crude and adjusted hazard ratios for all-cause mortality according to dialysis method.

HR of dialysis methods (HD vs. PD)	Univariate HR (95% CI)	*P*	Multivariate model 1^a^ HR (95% CI)	*P*	Multivariate model 2^b^ HR (95% CI)	*P*	Stepwise model HR (95% CI)	*P*
**All patients**								
Entire group (HD:156, PD:121)	1.17 (0.77–1.78)	0.46	0.92 (0.58–1.45)	0.72	0.95 (0.60–1.52)	0.84	1.10 (0.69–1.75)	0.68^c^
After PSM (HD:121, PD:121)	1.12 (0.72–1.75)	0.62	0.94 (0.59–1.49)	0.78	1.00 (0.62–1.62)	0.99	1.18 (0.73–1.89)	0.51^c^
**Younger than 65 years old patients**								
Entire group (HD:95, PD:88)	1.84 (1.01–3.34)	<0.05	1.79 (0.96–3.33)	0.07	2.35 (1.19–4.65)	<0.05	3.03 (1.48–6.20)	<0.05^d^
After PSM (HD:88, PD:88)	1.77 (0.96–3.24)	0.07	1.75 (0.94–3.27)	0.08	2.48 (1.23–4.99)	<0.05	3.28 (1.56–6.90)	<0.05^d^
**Older than 65 years old patients**								
Entire group (HD:61, PD:33)	0.46 (0.25–0.85)	<0.05	0.40 (0.20–0.78)	<0.05	0.28 (0.14–0.57)	<0.05	0.36 (0.19–0.69)	<0.05^e^
After PSM (HD:33, PD:33)	0.58 (0.29–1.15)	0.12	0.53 (0.26–1.10)	0.09	0.39 (0.17–0.87)	<0.05	0.37 (0.17–0.78)	<0.05^e^

PSM, propensity score matching; HR, hazard ratio; 95% CI, 95% confidence interval; PD, peritoneal dialysis; HD, hemodialysis; Cox’s multivariate regression model cofounders. ^a^Sex, age at dialysis initiation and Charlson Comorbidities Index (CCI). ^b^Sex, age at dialysis initiation, CCI, body mass index (BMI), left ventricular ejection fraction (LVEF), prealbumin (PA) and hemoglobin (HB). ^c^Cardiovascular disease (CAD), LVEF, age at dialysis initiation, low-density lipoprotein cholesterol (LDLC), high-density lipoprotein cholesterol (HDLC), BMI and diabetes. ^d^CAD, Cholesterol (TC), HDLC, LVEF, diabetes and BMI. ^e^LVEF and BMI.

For the short-term follow-up, the HR of all-cause mortality of HD tended to be higher than that of PD in the ≤65-year-old subgroup; otherwise, the HR of HD tended to be lower than that of PD in the >65-year-old subgroup but did not reach statistical significance (Supplementary Tables 1, 2).

For the long-term follow-up, the HR of all-cause mortality of HD tended to be higher than that of PD in the ≤65-year-old subgroup, but the HR of HD was lower than that of PD in the >65-year-old subgroup. In the univariate model, the HR (95% CI) of mortality for HD vs. PD was 0.41 (95% CI: 0.17–0.99), and in the fully adjusted multivariate model, it was 0.21 (95% CI: 0.08–0.58) (Supplementary Tables 1, 2).

For the HFrEF subgroup, the differences in mortality in all age subgroups disappeared (Tables 3, 5). For the HFpEF subgroup, the disadvantage of HD in the younger subgroup and its advantage in the elderly subgroup was preserved. The all-cause mortality HR of HD vs. PD for the younger subgroup was 3.06 (95% CI: 1.06–8.82) in multivariate model, while for the older subgroup was 0.32 (95% CI: 0.13–0.81) in multivariate model, and the HR remained significant both in the stepwise model and after PSM (Tables 3, 6).

**TABLE 5:** Crude and adjusted hazard ratios for all-cause mortality with HFrEF (LVEF ≤ 55%) according to dialysis method.

HR of dialysis methods (HD vs PD)	Univariate HR (95% CI)	*P*	Multivariate model 1^a^ HR (95% CI)	*P*	Multivariate model 2^b^ HR (95% CI)	*P*	Stepwise model HR (95% CI)	*P*
**All patients**								
Entire group (HD:43, PD:34)	1.16 (0.60–2.23)	0.65	0.98 (0.46–2.07)	0.96	1.08 (0.50–2.31)	0.84	2.08 (0.99–4.36)	0.05^c^
After PSM (HD:34, PD:34)	1.12 (0.56–2.24)	0.76	1.20 (0.54–2.64)	0.65	1.54 (0.65–3.65)	0.32	2.04 (0.92–4.53)	0.08^c^
**Younger than 65 years old patients**								
Entire group (HD:25, PD:30)	1.03 (0.42–2.48)	0.96	1.45 (0.57–3.66)	0.44	1.43 (0.51–4.02)	0.50	5.68 (1.46–22.08)	<0.05^d^
After PSM (HD:25, PD:25)	0.92 (0.37–2.28)	0.86	1.47 (0.56–3.87)	0.44	1.18 (0.38–3.70)	0.78	4.70 (1.26–17.54)	<0.05^d^
**Older than 65 years old patients**								
Entire group (HD:18, PD:4)	0.50 (0.16–1.56)	0.23	0.37 (0.10–1.34)	0.13	0.52 (0.11–2.56)	0.43	0.54 (0.17–1.78)	0.31^e^
After PSM (HD:4, PD:4)	0.86 (0.19–3.92)	0.84	0.20 (0.02–2.51)	0.21	–^f^	–^f^	4.52 (0.20–101.47)	0.34^e^

HFrEF, heart failure with reduced left ventricular ejection fraction; PSM, propensity score matching; HR, hazard ratio; 95% CI, 95% confidence interval; PD, peritoneal dialysis; HD, hemodialysis. Cox’s multivariate regression model cofounders. ^a^Sex, age at dialysis initiation and Charlson Comorbidities Index (CCI). ^b^Sex, age at dialysis initiation, CCI, body mass index (BMI), left ventricular ejection fraction (LVEF), prealbumin (PA) and hemoglobin (HB). ^c^Triglyceride (TG), Phosphorus (P) and plasma albumin (ALB). ^d^TG, P, Calcium (Ca), sex and LVEF. ^e^TG and HB. ^f^The confidence interval is too large due to the small sample size.

**TABLE 6:** Crude and adjusted hazard ratios for all-cause mortality with HFpEF (LVEF > 55%) according to dialysis method.

HR of dialysis methods (HD vs PD)	Univariate HR (95% CI)	*P*	Multivariate model 1^a^ HR (95% CI)	*P*	Multivariate model 2^b^ HR (95% CI)	*P*	Stepwise model HR (95% CI)	*P*
**All patients**								
Entire group (HD:113, PD:87)	1.23 (0.71–2.13)	0.46	0.96 (0.53–1.73)	0.88	0.97 (0.52–1.83)	0.93	0.88 (0.49–1.58)	0.66^c^
After PSM (HD:87, PD:87)	1.21 (0.68–2.16)	0.52	1.01 (0.55–1.83)	0.98	1.13 (0.59–2.14)	0.71	1.14 (0.61–2.11)	0.68^c^
**Younger than 65 years old patients**								
Entire group (HD:70, PD:58)	3.83 (1.46–10.05)	<0.05	3.31 (1.21–9.03)	<0.05	3.06 (1.06–8.82)	<0.05	2.98 (1.04–8.54)	<0.05^d^
After PSM (HD:58, PD:58)	3.19 (1.18–8.64)	<0.05	3.06 (1.10–8.49)	<0.05	3.08 (1.05–9.06)	<0.05	3.39 (1.12–10.24)	<0.05^d^
**Older than 65 years old patients**								
Entire group (HD:43, PD:29)	0.33 (0.15–0.72)	<0.05	0.29 (0.12–0.69)	<0.05	0.32 (0.13–0.81)	<0.05	0.23 (0.09–0.59)	<0.05^e^
After PSM (HD:29, PD:29)	0.26 (0.10–0.68)	<0.05	0.24 (0.09–0.67)	<0.05	0.29 (0.10–0.82)	<0.05	0.24 (0.08–0.70)	<0.05^e^

HFpEF, heart failure with preserved left ventricular ejection fraction; PSM, propensity score matching; HR, hazard ratio; 95% CI, 95% confidence interval; PD, peritoneal dialysis; HD, hemodialysis. Cox’s multivariate regression model cofounders. ^a^Sex, age at dialysis initiation and Charlson Comorbidities Index (CCI). ^b^Sex, age at dialysis initiation, CCI, body mass index (BMI), left ventricular ejection fraction (LVEF), prealbumin (PA) and hemoglobin (HB). ^c^Cardiovascular disease (CAD), age at dialysis initiation, LVEF, high-density lipoprotein cholesterol (HDLC), BMI and diabetes, low-density lipoprotein cholesterol (LDLC). ^d^CAD, diabetes, LVEF, BMI and PA. ^e^BMI, HDLC, age at dialysis initiation, HB, TG, and TC.

### Cause of death

With regard to the cause of death, among all age groups, the proportions of infection-related deaths and cardiovascular deaths were basically similar. The proportion of cerebral hemorrhage was higher in the HD group in all age groups, but did not reach statistical significance.

We also used a competing risk model to analyse the hazard ratios for cause-specific death in patients receiving different dialysis modalities. We found that HD was a protective factor for infection-related death in the entire HFpEF population. The infectious mortality HR of HD vs. PD was 0.24 (95% CI: 0.06–0.93) in univariate model and 0.12 (95% CI: 0.03–0.47) in multivariate model. The protective effect disappeared in young dialysis patients, but was evident in the elderly. The HR was 0.15 (95% CI: 0.03–0.68) in univariate model and 0.03 (95% CI: 0.00–0.36) in multivariate model (Table 7).

**TABLE 7:** Crude and adjusted hazard ratios for specific cause of mortality in competing risk model according to dialysis method.

	Cardiovascular and cerebrovascular mortality				Infection mortality			
		
HR of dialysis methods (HD vs PD)	Univariate HR (95% CI)	*P*	Multivariate^a^ HR (95% CI)	*P*	Univariate HR (95% CI)	*P*	Multivariate^a^ HR (95% CI)	*P*
**All patients**								
Entire group (HD:156, PD:121)	1.69 (0.87–3.29)	0.12	1.47 (0.65–3.32)	0.35	0.78 (0.32–1.88)	0.58	0.48 (0.18–1.25)	0.13
Reduced LVEF (≤55%) (HD:43, PD:34)	1.08 (0.42–2.75)	0.88	0.66 (0.21–2.11)	0.48	2.99 (0.64–13.92)	0.16	2.27 (0.37–13.95)	0.38
Preserved LVEF (>55%) (HD:113, PD:87)	2.61 (0.98–6.94)	0.06	2.68 (0.85–8.49)	0.09	0.24 (0.06–0.93)	<0.05	0.12 (0.03–0.47)	<0.05
**Younger than 65 years old patients**								
Entire group (HD:95, PD:88)	2.41 (0.96–6.06)	0.06	2.18 (0.72–6.60)	0.17	0.53 (0.09–3.35)	0.50	0.48 (0.06–3.65)	0.48
Reduced LVEF (≤ 55%) (HD:25, PD:30)	1.26 (0.34–4.72)	0.73	2.18 (0.74–6.48)	0.16	1.16 (0.07–18.08)	0.91	1.64 (0.15–18.40)	0.69
Preserved LVEF (> 55%) (HD:70, PD:58)	4.77 (1.08–21.11)	<0.05	4.43 (0.78–25.20)	0.09	0.34 (0.03–3.95)	0.39	0.19 (0.01–3.30)	0.25
**Older than 65 years old patients**								
Entire group (HD:61, PD:33)	0.97 (0.37–2.50)	0.94	0.90 (0.28–2.89)	0.86	0.58 (0.21–1.60)	0.29	0.47 (0.14–1.53)	0.21
Reduced LVEF (≤55%) (HD:18, PD:4)	0.41 (0.13–1.30)	0.13	0.35 (0.09–1.32)	0.12	1.51 (0.15–15.03)	0.73	2.05 (0.13–31.75)	0.61
Preserved LVEF (> 55%) (HD:43, PD:29)	1.29 (0.34–4.86)	0.70	1.41 (0.25–7.95)	0.69	0.15 (0.03–0.68)	<0.05	0.03 (0.00–0.36)	<0.05

HR, hazard ratio; 95% CI, 95% confidence interval; PD, peritoneal dialysis; HD, hemodialysis; LVEF, left ventricular ejection fraction. Competing risk multivariate model cofounders. ^a^Sex, age at dialysis initiation and Charlson Comorbidities Index (CCI).

## Discussion

We found that among ESRD patients with CHF younger than 65 years, the prognosis of those receiving PD was better than that of patients receiving HD. In contrast, among elderly patients, the prognosis of HD patients was better than that of PD patients. This difference in prognosis remained stable after multivariate analysis and PSM, but could not be reproduced in the subgroup of HFrEF (Figure 6 and Table 5). Compared with previous studies, our study once again confirmed the survival advantage of total HD in elderly patients (4). On the other hand, before our study (18), we did not find any research that reported a survival advantage of PD over HD for young ESRD patients with CHF, especially in patients with HFpEF (Figure 7 and Table 6) (14). In other words, both the survival advantage of PD in young adults and the survival advantage of HD in older adults are limited to the HFpEF population. These findings are meaningful for Chinese ESRD patients and clinicians in choosing a dialysis method.

Previous studies (19, 24) have shown that the survival rate of PD is higher in the first 2 years of dialysis, and the survival advantage of PD disappears after 2 years of dialysis. However, our study did not find such a situation. Our cohort did not find differences in survival between dialysis modalities in the first 2 years after initiation of dialysis or 2 years later. The hazard ratios for HD and PD were stable at different periods of follow-up.

We also observed a trend of differences in the proportions of cardiovascular deaths and cerebral hemorrhage deaths between the PD group and the HD group in the different age groups. However, we consider this to be related to the small scale of this study. With the extension of follow-up time and an increase in the number of patients, we have the opportunity to confirm the difference in the composition of the cause of death in future observations.

Our research is similar to that of Sens Florence in Sens et al. (2). Both studies showed that in the elderly subgroup, HD had a survival advantage over PD. However, in the younger group, the results of the two studies were opposite. Sens’s study suggested that the prognosis of HD was also better in the group younger than 75 years. However, our study found that the prognosis of PD was better in patients younger than 65 years.

The average age of patients in Sens’ study was significantly higher than that in our study. Moreover, the proportion of chronic glomerulonephritis as the primary disease of renal failure was significantly less than that of our patients (5.3–6.8 vs. 18.59–28.93%). We speculate that this is related to the low prevalence of kidney transplantation in China. Age is a strong factor in the death of dialysis patients. We have reason to believe that in young PD patients, the differences in the composition of the primary disease and the age at onset of dialysis is the reason for the difference in prognosis.

Another reason for the difference in prognosis may be the difference in the prevalence of cardiovascular disease. From the baseline characteristics, the proportion of cardiovascular diseases in the study by Sens fluctuated (41.2–43.4%), but our overall proportion of cardiovascular diseases was 27.44%. Moreover, in the ≤65-year-old subgroup, the proportion of cardiovascular disease in PD patients was particularly low (5.68%). However, among the >65-year-old subgroup, the proportion of cardiovascular disease (42.55%) was basically consistent with studies in France (2) and the United States (3).

Taking into account the problems noted in the previously mentioned French study (25), our study tried to collect objective indicators to accurately assess the patient’s cardiac function, such as the LVEF in echocardiography and the NYHA heart functional classification. Although retrospective studies do not guarantee timely recording of these data at the start of dialysis, we used data as close as possible to the start of follow-up. We compared the reported LVEFs and NYHA classifications of these patients and found no difference in heart function between those receiving HD and PD in each subgroup. Therefore, we believe that the cardiac function of the patients in each group was similar when they entered renal replacement therapy. In addition, according to our clinical experience, the cardiac function of dialysis patients changes dynamically throughout the dialysis process. Therefore, a baseline cardiac function evaluation alone cannot reflect the patient’s exposure to the central debilitating state during the entire dialysis treatment process. The time-dependent model needs to be followed up for further analysis.

Why does PD have an advantage in young CHF and ESRD patients? PD is recommended for patients with refractory heart failure who are not sensitive to diuretics (26). Possible mechanisms by which PD improves the prognosis of patients with heart failure include providing stable and continuous ultrafiltration, having minimal hemodynamic impact and eliminating a larger amount of sodium ions (27). On the other hand, HD was related to heart failure as a cause for hospitalization (28, 29). Recent studies have suggested that the use of icodextrin can improve the prognosis of patients with heart failure and that the main mechanism should be to improve the ultrafiltration of patients on PD (29). However, in this cohort, icodextrin was not yet widely used. This shows that, other than the better prognosis of our patients, the reason may not be directly related to the ultrafiltration advantage provided by icodextrin. Studies had shown that PD ultrafiltration can improve LVEF in HFrEF patients (30), thereby improving patient prognosis. We did not find differences in survival between the two dialysis modalities across age subgroups in HFrEF. This suggests that the survival advantage of PD in younger patients may be related to the survival advantage in HFpEF patients. The reasons for this phenomenon require further study.

In this study, among all dialysis patients ≤65 years, the average age of those receiving PD was lower; moreover, the proportion of patients with cerebrovascular diseases was lower because the choice of PD mostly relied on the patients themselves. It is difficult for patients with severe cerebrovascular diseases to receive PD for a long time. Therefore, the better prognosis of PD among young people may be related to fewer cerebrovascular complications at dialysis initiation.

To explore the reasons for the superior prognosis of young PD patients, we further analyzed the causes of death in the two groups of patients. The proportion of deaths from infections was basically the same between young and old patients (Table 1). However, when we compared the risk of death from infection in patients receiving the two dialysis modalities using a competing risk model, we found that patients receiving HD had a lower risk of death from infection, and HD mainly protected the elderly (Table 7).

Peritoneal dialysis inherently increases the risk of peritonitis (31, 32), although HD patients may die from blood access-related infections. However, pneumonia was responsible for the majority of infectious deaths. We therefore speculate that this is related to the treatment patterns of PD and HD. Unlike maintaining PD at home in elderly patients, elderly patients receiving HD visit the hospital at least twice a week and are treated by medical staff. This model enables more timely detection and intervention of potential infectious diseases, which may significantly reduce the risk of death from infection. In the future, we can test our hypothesis by analyzing the number of hospitalizations for infection and the severity of inflammation in patients with different dialysis methods.

On the other hand, PD patients are generally less likely to die from cerebral hemorrhage (Table 1). Due to the low incidence of events of interest and the large number of competing risk events, the variance of the hazard ratios of HD compared with PD patients for intracerebral hemorrhage death calculated by the competing risk model was very large, so it cannot be confirmed that HD is a risk factor for death from cerebral hemorrhage. Therefore, we considered cardiac and cerebrovascular events together (Table 7). However, we believe that with the extension of follow-up time and the accumulation of events of interest, the risk of HD for intracranial hemorrhage can be confirmed by a competing risk model. The most likely explanation is that heparin is necessary during HD. In addition, compared with elderly patients, younger patients have a higher rate of death from cerebral hemorrhage. The possible reason is the rapid fluctuation of blood pressure caused by the rapid change in volume during HD (26), which may also be a factor that increases the risk of cerebral hemorrhage. In our clinical experience, it is more feasible in young HD patients to prescribe high ultrafiltration rates after increased interdialytic weight gains to solve the volume overload compared to older patients, which will increase blood pressure fluctuations during the dialysis interval, more easily inducing cerebral hemorrhage.

The incidence of cardiovascular death among young PD patients is relatively low, and the incidence of cardiovascular events in elderly PD patients is significantly higher (Table 3). First, age is the most important factor influencing cardiovascular events. Among the subgroups ≤65 years old, the age of PD patients was significantly lower. A younger age is associated with less coronary atherosclerosis, which in turn is associated with less cardiovascular death. However, we understand that, of course, our research cannot provide relevant evidence. The quality of volume control is an important factor that affects the prognosis of dialysis patients (33). According to the mechanism of PD, the ultrafiltration of PD is relatively gentle, which also means that it takes a longer time for the volume to reach a suitable state than with HD. The characteristics of gentle volume fluctuations may have different effects on patients of different ages. Young patients with a strong self-management ability can maintain a reasonable volume for a long time, thus protecting heart function (34). Elderly patients have a weaker ability to self-manage and may have an insufficient or overloaded volume that cannot be corrected in a timely manner; therefore, it is detrimental to the protection of cardiac function (35).

Our previous studies have found that among dialysis-dependent patients less than or equal to 60 years old, the prognosis of PD is better than that of HD. We do not know the reason for the survival advantage of PD. Similarly, this study found a survival advantage of PD in patients younger than 65 years by analysing the survival of ESRD patients with CHF. Moreover, the proportion of deaths from cardiovascular diseases among young people is relatively low. These phenomena indicate that PD may have certain advantages in reducing cardiovascular events in young patients. In turn, PD has a survival advantage in young dialysis-dependent patients.

### Advantages

This article compares the prognosis between ESRD patients with CHF receiving HD and those receiving PD in southern China for the first time. We have a larger number of follow-ups, and the follow-up time is longer than that in previous studies. This research can truly reflect the actual situation of dialysis patients in our center. Compared with our previous research, this time, we completed the investigation of the cause of death and explained to a certain extent the reason for the difference in survival between the two dialysis methods. We identified the cause of death of each patient by consulting the death certificate registration data of the Chinese CDC, which is a relatively objective and accurate method.

In the current situation where kidney transplantation has a low prevalence and is expensive (36), our study provides a reference for Chinese ESRD patients and nephrologists in choosing a dialysis method.

### Limitations

The limitations of retrospective research need to be clearly recognized. One is selection bias. There are many factors that affect patient selection of dialysis methods. Long-term prognosis is one of them, but medical insurance policies and personal economic conditions may be larger factors. With the expansion of the follow-up population and the extension of the follow-up time, the conclusions of this article may be overturned.

There were some important baseline data, such as the delay between the diagnosis of CHF and the start of dialysis or the volume of residual diuresis, that were incomplete in this study, which leads to our imperfect evaluation of the patients’ baseline cardiac and residual kidney function. On the other hand, the cardiac function of dialysis patients continues to change over the treatment duration. Regardless of whether multivariate Cox regression or PSM was used, only the patient’s baseline cardiac function was considered. Therefore, follow-up studies can use the time-dependent Cox regression model (37) to evaluate in detail the impact of changes in cardiac function on the prognosis of dialysis patients. The survival differences could not be reproduced in the subgroup of HFrEF (Figure 6). These results could not be extrapolated to the population of patients with ESKD and HFrEF.

The investigation of the cause of death in this study did not originate from autopsy, and the cause of death of some patients was learned through interviews with patients’ family members after death. Therefore, the speculation of the cause of death may not be accurate. In particular, the proportion of cardiovascular deaths and cerebrovascular deaths may be underestimated. Notably, this underestimation may be completely randomly distributed, so it should be equivalent for both dialysis methods.

We found that almost no PD patients died of cerebral hemorrhage or bleeding disorders. However, due to our small number of cases, the HR confidence interval derived from the competing risk model is too large to be show in the article. We hypothesized that HD patients should be more likely to die from intracerebral hemorrhage than PD patients, which requires further study.

## Conclusion

This study suggests that PD may have a better prognosis for young ESRD patients with CHF and preserved LVEF in southern China. For elderly patients, the prognosis of HD is better.

## Data availability statement

The raw data supporting the conclusions of this article will be made available by the authors, without undue reservation.

## Ethics statement

The studies involving human participants were reviewed and approved by Ethics Committee of Guangdong Provincial Hospital of Chinese Medicine. Written informed consent for participation was not required for this study in accordance with the national legislation and the institutional requirements.

## Author contributions

ZH, HH, XL, and LW contributed to conception and design of the study. ZH, HL, JH, DZ, LZW, FY, and DXZ organized the database. ZH, HCL, and WQ performed the statistical analysis. ZH wrote the first draft of the manuscript. HM, JL, YC, and TL wrote sections of the manuscript. All authors contributed to manuscript revision, read, and approved the submitted version.
