# Supplementary material for: Impact of dialysis modality choice on the survival of end-stage renal disease patients with congestive heart failure in southern China: A retrospective cohort study
Source: Front Med (Lausanne). 2022 Oct 17;9:898650. doi: 10.3389/fmed.2022.898650 (PMC9623394; doi:10.3389/fmed.2022.898650)
Supplement: Supplementary file 1 [file Table_1.DOCX]

| Table 1 Harard ratio of mortality assessed by univariate and multivariate Cox’s proportional hazards model. | | | | | | | |
| --- | --- | --- | --- | --- | --- | --- | --- |
| HR of dialysis modality  (HD vs PD) | Univariate  HR(95% CI) | Multivariate  model 1^a^  HR(95% CI) | Multivariate  model 2^b^  HR(95% CI) | Multivariate  model 3^c^  HR(95% CI) | Multivariate  model 4^d^  HR(95% CI) | Multivariate  model 5^e^  HR(95% CI) | Multivariate  model 6^f^  HR(95% CI) |
| All patients | | | | | | | |
| Entire follow-up period  (HD:156, PD:121) | 1.17(0.77-1.78) | 1.22(0.79-1.87) | 1.18(0.76-1.82) | 1.22(0.79-1.90) | 1.08(0.69-1.69) | 1.03(0.66-1.62) | 1.06(0.68-1.67) |
| First 2 years follow-up  (HD:156, PD:121) | 1.13(0.56-2.27) | 1.19(0.59-2.43) | 1.14(0.56-2.33) | 1.27(0.61-2.62) | 1.16(0.56-2.40) | 1.12(0.54-2.32) | 1.12(0.54-2.32) |
| After first 2 years follow-up  (HD:121, PD:82) | 1.20(0.71-2.03) | 1.23(0.72-2.11) | 1.20(0.69-2.07) | 1.21(0.70-2.10) | 1.04(0.59-1.86) | 1.00(0.56-1.77) | 1.03(0.58-1.84) |
| Younger than 65 years old patients | | | | | | | |
| Entire follow-up period  (HD:95, PD:88) | **1.84(1.01-3.34)** | **1.99(1.09-3.65)** | **2.00(1.09-3.68)** | **2.09(1.11-3.95)** | **1.95(1.03-3.70)** | **1.93(1.02-3.65)** | **2.26(1.16-4.43)** |
| First 2 years follow-up  (HD:95, PD:88) | 2.74(0.74-10.12) | 3.00(0.80-11.19) | 2.87(0.77-10.72) | 3.20(0.83-12.31) | 3.11(0.80-12.10) | 2.81(0.72-11.01) | 3.54(0.86-14.63) |
| After first 2 years follow-up  (HD:74, PD:67) | 1.63(0.83-3.21) | 1.75(0.88-3.48) | 1.83(0.91-3.67) | 1.88(0.91-3.89) | 1.71(0.83-3.57) | 1.71(0.83-3.55) | 1.93(0.90-4.15) |
| Older then 65 years old patients | | | | | | | |
| Entire follow-up period  (HD:61, PD:33) | **0.46(0.25-0.85)** | **0.45(0.23-0.85)** | **0.47(0.24-0.91)** | **0.47(0.25-0.90)** | **0.48(0.25-0.91)** | **0.48(0.25-0.90)** | **0.32(0.16-0.61)** |
| First 2 years follow-up  (HD:61, PD:33) | 0.50(0.21-1.19) | 0.53(0.22-1.30) | 0.57(0.23-1.42) | 0.60(0.24-1.48) | 0.60(0.24-1.47) | 0.58(0.24-1.43) | **0.37(0.15-0.92)** |
| After first 2 years follow-up  (HD:47, PD:15) | **0.41(0.17-0.99)** | **0.37(0.15-0.92)** | **0.36(0.14-0.92)** | **0.36(0.14-0.91)** | **0.36(0.14-0.91)** | **0.36(0.14-0.91)** | **0.21(0.08-0.58)** |

1. HR, hazard ratio; 95% CI, 95% confidence interval; PD, peritoneal dialysis; HD, hemodialysis; LVEF, left ventricular ejection fraction.
2. Cox’s multivariate regression model cofounders: ^a^ sex. ^b^ sex and cardiovascular disease (CAD). ^c^ sex, CAD and Charlson Comorbidities Index (CCI). ^d^ sex, CAD, CCI and body mass index (BMI). ^e^ sex, cardiovascular disease, CCI, BMI, prealbumin (PA), high-density lipoprotein cholesterol (HDLC) and hemoglobin (Hb). ^f^ sex, cardiovascular disease, CCI, BMI, PA, HDLC, Hb and LVEF.

| Table 2 Harard ratio of mortality assessed by univariate and multivariate Cox proportional hazards model after propensity score matching. | | | | | | | |
| --- | --- | --- | --- | --- | --- | --- | --- |
| HR of dialysis methods  (HD vs PD) | Univariate  HR(95% CI) | Multivariate  model 1^a^  HR(95% CI) | Multivariate  model 2^b^  HR(95% CI) | Multivariate  model 3^c^  HR(95% CI) | Multivariate  model 4^d^  HR(95% CI) | Multivariate  model 5^e^  HR(95% CI) | Multivariate  model 6^f^  HR(95% CI) |
| All patients | | | | | | | |
| Entire follow-up period  (HD:121, PD:121) | 1.12(0.72-1.75) | 1.15(0.73-1.80) | 1.20(0.76-1.88) | 1.25(0.79-1.97) | 1.13(0.71-1.80) | 1.11(0.70-1.77) | 1.11(0.70-1.76) |
| First 2 years follow-up  (HD:121, PD:121) | 1.12(0.53-2.35) | 1.14(0.54-2.39) | 1.15(0.55-2.42) | 1.32(0.62-2.81) | 1.23(0.58-2.61) | 1.20(0.56-2.56) | 1.17(0.54-2.50) |
| After first 2 years follow-up  (HD:94, PD:82) | 1.12(0.64-1.96) | 1.15(0.66-2.03) | 1.23(0.70-2.16) | 1.24(0.70-2.19) | 1.10(0.61-1.97) | 1.08(0.60-1.95) | 1.09(0.61-1.96) |
| Younger than 65 years old patients | | | | | | | |
| Entire follow-up period  (HD:88, PD:88) | 1.77(0.96-3.24) | **1.91(1.03-3.53)** | **1.94(1.05-3.60)** | **2.13(1.12-4.07)** | **2.02(1.05-3.86)** | **2.03(1.06-3.89)** | **2.41(1.21-4.78)** |
| First 2 years follow-up  (HD:88, PD:88) | 2.64(0.70-9.96) | 2.86(0.76-10.85) | 2.80(0.74-10.62) | 3.15(0.80-12.38) | 3.12(0.79-12.34) | 2.78(0.70-11.15) | 3.37(0.80-14.09) |
| After first 2 years follow-up  (HD:69, PD:67) | 1.57(0.79-3.11) | 1.69(0.84-3.38) | 1.80(0.89-3.64) | 1.96(0.93-4.12) | 1.80(0.85-3.80) | 1.84(0.88-3.88) | 2.11(0.96-4.63) |
| Older then 65 years old patients | | | | | | | |
| Entire follow-up period  (HD:33, PD:33) | 0.58(0.29-1.15) | 0.57(0.28-1.14) | 0.65(0.32-1.34) | 0.68(0.34-1.37) | 0.69(0.34-1.39) | 0.72(0.35-1.47) | **0.37(0.17-0.79)** |
| First 2 years follow-up  (HD:33, PD:33) | 0.63(0.24-1.67) | 0.63(0.24-1.66) | 0.71(0.26-1.92) | 0.75(0.28-2.01) | 0.74(0.28-2.00) | 0.81(0.30-2.18) | 0.51(0.17-1.54) |
| After first 2 years follow-up  (HD:24, PD:15) | 0.53(0.20-1.39) | 0.52(0.19-1.38) | 0.55(0.19-1.56) | 0.56(0.20-1.58) | 0.57(0.20-1.63) | 0.52(0.18-1.55) | **0.26(0.08-0.83)** |

1. PSM, Propensity score matching; HR, hazard ratio; 95% CI, 95% confidence interval; PD, Peritoneal dialysis; HD, Hemodialysis; LVEF, left ventricular ejection fraction.
2. Cox’s multivariate regression model cofounders: ^a^ sex. ^b^ sex and cardiovascular disease (CAD). ^c^ sex, CAD and Charlson Comorbidities Index (CCI). ^d^ sex, CAD, CCI and body mass index (BMI). ^e^ sex, cardiovascular disease, CCI, BMI, prealbumin (PA), high-density lipoprotein cholesterol (HDLC) and hemoglobin (Hb). ^f^ sex, cardiovascular disease, CCI, BMI, PA, HDLC, Hb and LVEF.
3. PSM covariates of all groups: sex, age of dialysis initiation and CCI.
